# Prospective of Essential Oils of the Genus *Mentha* as Biopesticides: A Review

**DOI:** 10.3389/fpls.2018.01295

**Published:** 2018-09-10

**Authors:** Pooja Singh, Abhay K. Pandey

**Affiliations:** Bacteriology and Natural Pesticide Laboratory, Department of Botany, DDU Gorakhpur University, Gorakhpur, India

**Keywords:** *Mentha*, essential oil, antimicrobial, chemical composition, insecticidal

## Abstract

*Mentha* is a genus from the family Lamiaceae, whose essential oils has long been used in various forms such as in management of plant pathogens and insect pests, in traditional medicine as well as in culinary and cosmetics. Its major chemical components such as menthol, carvone have now been successfully commercialized in the industry as antimicrobials/insecticidal agents. Current review focuses on chemical composition of essential oils of some *Mentha* species from different geographical regions with their insecticidal (repellent, antifeedant, and ovicidal) and antimicrobial efficacies against bacterial, fungal plant pathogens and insects of stored products. Reports of the researchers on chemical analysis of essential oils of *Mentha* species revealed that most of the oils being rich in pulegone, menthon, menthol, carvone, 1, 8-cineole, limonene and β-caryophyllene. Reviewed literature revealed that, essential oils from different *Mentha* species possess potential antimicrobial activity against plant pathogens and have insecticidal activity against stored product insects. Thus, antimicrobial and insecticidal properties of essential oils of *Mentha* species offer the prospect of using them as natural pesticides with a commercial value, having social acceptance due to its sustainability and being environment friendly.

## Introduction

In the plant kingdom, family Lamiaceae (syn. Labiatae) is endowed with several medicinal and aromatic plants. The family comprises of more than 232 genera and approximately 7200 species (Harley et al., 2004). Most of the Lamiaceae plants are found to accumulate secondary metabolites such as terpenes/essential oils and other components, mainly in the epidermal glands of leaves, stems and reproductive structures. These terpenes/essential oils have several applications in cosmetics, food industry, medication and perfumery. The genus *Mentha* is a very important taxon in the family Lamiaceae and includes 25–30 species that grow worldwide especially in the South Africa, Australia and temperate regions of Eurasia (Dorman et al., 2003). The taxon has a significant importance both commercial as well as medicinal. Indeed, different plant parts such as leaves, flowers and stems of this genus are frequently used in herbal medicine, teas or as additives in spice mixtures for various foods to offer aroma and flavor (McKay and Blumberg, 2006). Additionally, *Mentha* spp. has been used as a folk remedy for aliment ulcerative, anorexia, nausea, flatulence, bronchitis, liver complaints, and colitis due to its stimulant, antiemetic, diaphoretic, carminative, antiinflammatory, analgesic, emmenagogue, antispasmodic, and anticatarrhal activities (Iscan et al., 2002; Moreno et al., 2002). Commercially, the most important mint species are spearmint (*Mentha spicata*), peppermint (*M. x piperita*), and corn mint (*M. canadensis*). Among these species, corn mint is only cultivated for oil production (Oudhia, 2003). Peppermint oil is also used for its essential oils and mostly because of its major components menthol and menthone (Mimica-Dukic et al., 2003). On the other hand spearmint is rich in carvone and is widely used as spices and cultivated in several countries (Kokkini et al., 1995). Peppermint oil is applied to flavor the pharmaceuticals and most of the oral preparations (toothpastes, dental creams, and mouth washes). The plant is also used as flavoring agent in confectionery, cough drops, chewing gums, and some of the alcoholic liqueurs. For internal uses it is used in medicines and its pleasant taste makes it an excellent gastric stimulant (Dorman et al., 2003). Due to increasing interest in tribal and traditional phyto-therapeutics methods, many recent investigations have been carried out to examine the medicinal properties of these herbs for human welfare. McKay and Blumberg (2006) published review on the bioactivity and potential health activity of *M. piperita*. Their studies were only focused on medicinal properties and they took only one plant species during the compilation. Kumar et al. (2010) reviewed insecticidal properties of *Mentha* oil and extract, but they only covered the insecticidal properties against storage insects. Still, nobody has compiled a review on antibacterial, antifungal activities and insecticidal activities of *Mentha* oil against plant pathogens/storage insect pests. Henceforth, in this review, we discuss latest advances in the chemistry, antimicrobial and insecticidal activities of essential oils from different *Mentha* species of different regions. The keywords used to survey the literature are Mentha essential oil, chemical composition, antifungal, antibacterial and insecticidal. For a comprehensive literature overview, we analyzed the published phytochemical and biological data available through several search engines, such as ^®^SciFinder, ISI ^®^Web of Science, ^®^Scopus, and ^®^Google Scholar, as well as several libraries viz., National Science Library, New Delhi; National Medical Library, New Delhi; IARI Library, New Delhi; CIMAP, Lucknow, and FRI Library, Dehradun of India.

## Essential Oil Composition of *Mentha* Species

**FIGURE 1:**
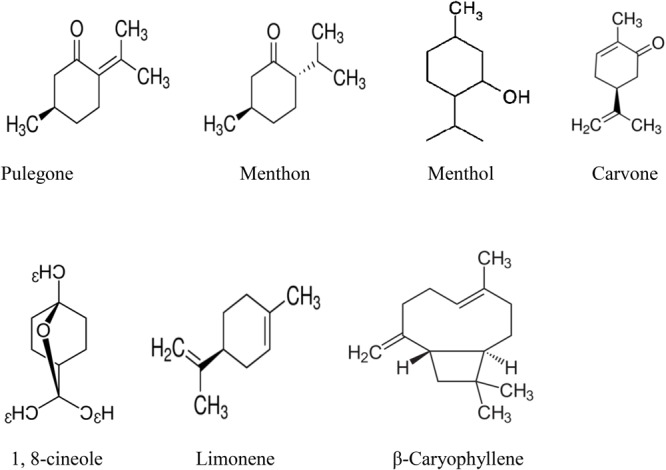
Active compounds of different species of *Mentha* essential oil.

Essential oils of higher plants are volatile in nature and a complex mixture of monoterpenes (C_10_) and sesquiterpenes (C_15_); although diterpenes (C_20_) may also be present, and a variety of low molecular weight aliphatic hydrocarbons, alcohols, acids, aldehydes, acyclic esters or lactones and exceptionally N- and S-containing compounds, coumarins and homologs of phenyl-propanoids (Dorman and Deans, 2000; Sharifi-Rad M. et al., 2017; Sharifi-Rad J. et al., 2017). The whole *Mentha* plant possesses essential oils; however, the amount of oil varied depending upon the species and method of isolation. Essential oils from different *Mentha* species have been isolated by different methods such as hydrodistillation using Clevenger apparatus or pharmacopeia distillation apparatus (Li et al., 2013). Menthol and pulegone present in the essential oil of *Mentha* species are the substances that give the mints their characteristic aromas and flavors (Lubbe and Verpoorte, 2011). Some investigators reported that aroma and flavor of spearmint is due to presence of carvone (Hussain et al., 2010a). Instead of menthol, pulegone and carvone, chemically essential oil of *Mentha* species is composed of other different major and minor components. Several investigations have been carried out on the chemical composition of different samples of *Mentha* species from different geographical regions. The *Mentha* species which are described here in **Table 1** for their essential oil chemistry have been collected from the different geographical regions. Most of the species were collected from Bangladesh, Brazil, Cameroon, Egypt, Europe, Guinea, India, Iran, Italy, Tunisia, Mali, Nigeria, Pakistan, Rwanda, Thailand, Togo, Turkey, Um Ruaba, and Yaounde. The studies revealed that chemical composition and their respective percentage of different *Mentha* species varied depending upon the origins of the plant and species (**Table 1**). Table shows that the species investigated for the chemical composition of the oil from different countries were *M. piperita, M. pulegium, M. longifolia, M. arvensis, M. suaveolens, M. rotundifolia, M. officinalis, M. spicata, M. mozaffarianii, M. x villoso-nervata, M. viridis*, and *M. rotundifolia.* **Table 1** also shows that there is a significant variation in chemical composition of the same species. This may be due to the time of oil extraction or their occurrence in different geographical location. The compositional variation in the essential oil is also may be due to harvesting time at different stage, drying as well as extraction methods (Rohloff et al., 2005). Some factor like physiological and environmental conditions, genetic and evolution also determine the chemical variability of *Mentha* essential oils (Figueiredo et al., 2008). Additionally, most of the species chemically characterized were rich in pulegone, menthon, menthol, carvone, 1, 8-cineole, limonene, and β-caryophyllene. The chemical structures of major compounds are depicted in **Figure 1**.

**Table 1:** Chemical composition of different species of the *Mentha* oil.

Plant species	Origin	Major component (%)	Researchers
*Mentha piperita*	–	Menthol (29–48), Menthone (20–31), Menthofuran (6.8), Menthyl acetate (3–10)	Clark and Menory, 1980
	Turkey, Jet-Farms, Yakima, Mari-Linn Farms, Erdogmus Perfume Industry, imported from India	Menthol (28–42), Menthone (18–28)	Iscan et al., 2002
	Iran	α-Terpinene (19.7), Isomenthone (10.3), *Trans-*carveol (14.5), Piperitinone oxide (19.3), β-Caryophyllene (7.6)	Yadegarinia et al., 2006
	Sri Lanka	Menthol (41.2), Menthone (24.3), β-Caryophyllene (5.1), Menthyl acetate (2.0), Limonene (1.1), α-Pinene (1.1)	Samarasekera et al., 2008
	Serbia	Menthol (37.4), Menthyl acetate (17.4), Menthone (12.7)	Soković et al., 2009
	Pakistan	Menthone (28.13 and 25.54), Menthyl acetate (9.51 and 9.68), limonene (7.58 and 7.73), isomenthone (4.04 and 7.63), summer and winter, respectively	Hussain et al., 2010a
	Faso	Menthol (39.3), Menthone (25.2), Menthofuran (6.8), Menthyl acetate (6.7), *iso*-Menthone (5.3), 1, 8-Cineole (4.1), Pulegone (1.4)	Bassolé et al., 2010
	Algeria	Menthol (33.28), Menthone (22.03), Menthyl acetate (6.40)	Djenane et al., 2012
	Iran	Menthol (53.28), Menthyl acetate (15.1), Menthofuran (11.18)	Saharkhiz et al., 2012
	China	Menthol (30.69), menthone (14.51) and menthy acetate (12.86),	Sun et al., 2014
	Algeria	Limonene oxide (23,3), followed by 7-Oxabicyclo[4,1,0]heptane,1-methyl-4-(methylethenyl)- (14,6), *Cis*-(-)-1,2-Epoxy-p-menth-8-ene (5,72), and Bicyclo[2.2.1] heptane-2,5-diol,1,7,7-trimethyl-,(2-endo,5-exo)-(4,04)	Mehani et al., 2015
	Brazil	Carvone (84.34) and limonene (10.97)	Rezende et al., 2017
*M. pulegium*	Aksu (Turkey)	Pulegone (205.5 mg/ml), 1,8-Cineole (34.7 mg/ml), Borneol (13.8 mg/ml), Menthone (5.4 mg/ml)	Müller-Riebau et al., 1995
	Mt. Pangaio (Greece)	Isomenthone (77.5), Menthone (10.3), Pulegone (1.0)	Sivropoulou et al., 1995
	Antalya, Termessus, Aksu, Düden and Kalkan (Turkey)	Pulegone (39.6–419.6 mg/ml), Menthone (12.2–166.0 mg/ml), borneol (16.7–47.6 mg/ml), 1,8 cineole (19.8–40.1 mg/ml)	Müller-Riebau et al., 1997
	Sintra (Portugal)	Pulegone (78.3–80.9), Menthone (8.5–9.2)	Reis-Vasco et al., 1999
	Uruguay	Pulegone (73.4), Isomenthone (12.9)	Lorenzo et al., 2002
	Moroccan	Pulegone (85.4)	Bouchra et al., 2003
	Bandar-e Anzali (Iran)	Pulegone (37.8), Menthone (20.3), Piperitone (6.8)	Aghel et al., 2004
	Jammu region and Kashmir valley (India)	Pulegone (65.9–83.1), Menthone (8.3–8.7), Isomenthone (3.8–4.0)	Agnihotri et al., 2005
	Bulgaria	Pulegone (42.9–45.4)	Stoyanova et al., 2005
	Egypt	Pulegone (43.5), Piperitone (12.2)	El-Ghorab, 2006
	Tunisia	Pulegone (17.5–70.2), Carvone (trace to 55.7), Isomenthone (2.9–34.2), Menthol (0.1–21.2), Menthofuran (0.7–10.0)	Mkaddem et al., 2007
	Portuguese market	Pulegone (35.1), Piperitenone (27.4)	Mata et al., 2007
	Algeria	Pulegone (4.4–87.3), Piperitenone (0.1–26.7), Isomenthone (trace to 22.6), α-Pinene (0.4–20.9)	Beghidja et al., 2007
	Spanish	Pulegone (41.1–42.3), Piperitone oxide (14.9–16.9), Piperitenone (4.6–6.1), Piperitone (5.4–6.0)	Díaz-Maroto et al., 2007
	Iran	Piperitone (38.0), Piperitenone (33.0), α-Terpineol (4.7), Pulegone (2.3)	Mahboubi and Haghi, 2008
	Tunisian	Menthol (48.56), Menthone (12.34), 1–8 Cineole (17.31), Pulegone (3.76)	Marzouk et al., 2008
	Samos, Argos, Evia, Samothraki, and Kalamata (Greece)	Pulegone (61.3–77.9), Iso-menthone (10.6–18.5), Menthone (0.6–8.3), Piperitone (0.3–3.2), *Cis*-isopulegone (0–1.7)	Petrakis et al., 2009
	Tunisia	Pulegone (61.11)	Hajlaoui et al., 2009
	Iran	Pulegone (40.5), Menthone (35.4), Piperitone (5.2)	Kamkar et al., 2010
	Turkey	(+)-Menthol (38.06), Menthol (35.64), Neomenthol (6.73), Cineole (3.62)	Kizil et al., 2010
	Morocco	Pulegone (70), Piperitenone (3.1), Isopulegone (1.8), Piperitone epoxide cis (1.7)	Ait-Ouazzou et al., 2011
	Europe (Portugal)	Menthone (35.9), Pulegone (23.2), Neo-menthol (9.2), 8-Hydroxy; sigma 4(5)-p-menthen-3-one (2.1)	Teixeira et al., 2012
	Portugal	Pulegone as the major compound (52–82), followed by isomenthone (2–36), menthone (0.1–17), and piperitenone (1–15%)	Rodrigues et al., 2013
	North Morocco	Pulegone (33.65) α-terpinenyl acetate (24.29), bicyclo[3.1.0]hexane, 6-isopropylidene-1-methyl-(12.59), 1,8-cineole (10.53), α-humulene (5.58) and α-pinene (5.34)	Cherrat et al., 2014
	Tunisian	Pulegone (61.11), Isomenthone (17.02), Piperitone (2.63)	Hajlaoui et al., 2009
*M. longifolia*	Topolia (Greece)	Carvone (58.0), *Trans*- and *cis*-dihydrocarvone (0.2–32.9)	Kokkini et al., 1995
		Piperitone, Menthone, Pulegone, Neo-menthol, Isomenthone	Mimica-Dukic et al., 2003
	Iran	*Cis-*carveol (53–78)	Zenali et al., 2005
	Pakistan	Piperitenone oxide (60.10 and 64.60), Piperitenone (6.37 and 1.97), Germacrene D (5.13 and 5.97) summer and winter, respectively	Hussain et al., 2010a
		*M. longifolia:* Pulegone (54.41), Isomenthone (12.02), 1,8-cineole (7.41), Borneol (6.85), Piperitenone oxide (3.19)	Mkaddem et al., 2009
	Turkey	*Cis*-piperitone epoxide (18.4), Pulegone (15.5), Piperitenone oxide (14.7), Menthone (7.9), Isomenthone (6.6), *Trans*-piperitone epoxide (4.1), Carvone (4.9)	Gulluce et al., 2007
	2 ecotypes (Sidi Bouzid and Gabes) from Tunisie	1,8-cineole (5.6–10.8), menthone (20.7–28.8), terpineol-4 (3.1–4.9), menthol (19.4–32.5), pulegone (7.8–17.8) and piperitone (2.2–3.3)	Hajlaoui et al., 2008
	Tunisie	Menthol (32.51), Menthone (20.71), Pulegone (17.76), 1,8-Cineole (5.61), Terpineol-4 (4.87), Piperitone (2.16)	Hajlaoui et al., 2010
	Sénégal	Pulegone (52.0 and 42.4), menthone (14.3 and 21.2), 1, 8-cineole (13.1 and 11.4) and isomenthone (9.0 and 13.2)	Diop et al., 2016
	Tunisian	Pulegone (47.15), 1, 8-Cineole (11.54), Menthone (10.7), α-Pinene (3.57), α-Terpineol (3.17), d-Cadinene (3.53)	Hajlaoui et al., 2009
	Central Greece, Southern Greece	Central Greece: Piperitenone oxide (33.4), 1,8-Cineole (24.5), *Trans*-piperitone epoxide (17.4) Southern Greece: Carvone (54.7), Limonene (20.0)	Koliopoulos et al., 2010
*M. longifolia* subsp. *polyadena*	South Africa	Menthofuran-rich type (51–62), *cis* Piperitone oxide (15–36), Piperitenone oxide-rich type (15–66)	Viljoen et al., 2006
*M. longifolia* (L.) L. subsp. *capensis* (Thunb.) Briq	South Africa	Menthone (50.9), Pulegone (19.3), 1,8-Cineole (11.9)	Oyedeji and Afolayan, 2006
*M. arvensis*	Austria	Menthone (24.0), Isomenthone (10.5), Neo-menthol (6.9), Menthol (33.5), Menthyl acetate (5.0)	Koschiera et al., 2002
	Punjab and Himachal Pradesh, India	L-Menthone , Menthol, Isomenthone, Eucalyptol, Piperitone oxide, Carvone, dl-Limonene, *trans*-Dihydrocarvone, Germacrene-D	Sharma et al., 2009
*M. suaveolens*	Greece	Piperitenone oxide (62.4)	Koliopoulos et al., 2010
*M. suaveolens* ssp. *Insularis*	France	Pulegone (44.4 and 14.8), *cis-cis-p-*Menthenolide (27.3 and 67.3)	Sutour et al., 2008
*M. rotundifolia*	Morocco	Menthol (40.50), Menthone (5.0), Menthyl acetate (4.50), Menthofuran (4.20), Oxyde de piperitone (3.80), Linalyl acetate (3.50), Neomenthol (3.20), Piperitone (3.10), Isomenthone (2.50), 1,8-Cineole (2.40), Linalool (2.0), Limonene (1.80), Geraniol (1.70), Myrcene (1.60), Geranyl acetate (1.50), *Trans*-Sabinene hydrate (1.40)	Derwich et al., 2010
	Tunisia	β-Caryophyllene (26.67), Germacrene D (12.31) and Carveol (7.38)	Riahi et al., 2013
*M. officinalis*	Greece	Terpin-4-ol (15.8), Caryophyllene oxide (13.2), Sabinene (12.9), β-Pinene (12.1), *Trans*-caryophyllene (10.2)	Koliopoulos et al., 2010
*M. spicata*	Soliman Tunisian	Carvone (40.8) and limonene (20.8)	Snoussi et al., 2015
	Greece	Piperitenone oxide (35.7), 1,8-Cineole (14.5)	Koliopoulos et al., 2010
	Pakistan	Carvone (59.50 and 63.24), Limonene (10.44 and 9.09), 1,8-cineol (6.36 and 4.51) summer and winter, respectively	Hussain et al., 2010b
	Serbia	Carvone (69.5) and Menthone (21.9)	Soković et al., 2009
*M. mozaffarian*	*Iran*	Piperitone (51.0)	Sam-Daliri et al., 2016
M. *x villoso-nervata*	Topolia	*M. x villoso-nervata*: Carvone (80.1), *Trans*- and *cis*-dihydrocarvone (0.1–5.4)	Kokkini et al., 1995
*M. viridis*		Carvone (50.47), 1,8-Cineole (9.14), Limonene (4.87)	Mkaddem et al., 2009
*M. rotundifolia*	Spain	Rotundifolone (10.4), Piperitol (57.6), Rotundifolone (33.2), Diosphenol (47.7)	Perez Raya et al., 1990

## Biological Activity of Essential Oils of *Mentha* Species

In recent years researchers have focused their attention on the biological activities of essential oils (EOs) and their constituents of different *Mentha* species from several research organizations of the world. Bacteria and fungi are important pathogens of the crops as well as stored food commodities; reported to cause 40–50% losses (Pandey et al., 2017). Here we have reviewed the activity of essential oils of different *Mentha* species against plant fungal pathogens and stored product insects, their mechanism as well as their applications in the postharvest.

### *Mentha* Essential Oil as an Antibacterial Agent

Bacteria associated with plants causes serious diseases in agricultural crops as well as in postharvest commodities throughout the world (Vidhyasekaran, 2002). The yield loss reported due to bacterial diseases in pre and postharvest is about 30–40% of crop produce per year. Basically, *Xanthomonas, Pseudomonas*, and *Erwinia* are the main genera of bacteria, play major role in causing plant diseases (Agrios, 2005). There are several commercial bactericides available in the markets against these bacterial pathogens, however, their indiscriminate use caused several health hazards to human beings as well side effect to the host plants (Louws et al., 2001). Additionally, there are several strains of bacterial pathogens which have developed resistance against commercial bactericides due to the change in climate. Plant bacteriologists reported that plant pathogenic bacteria readily acquire resistance to copper bactericides and streptomycin (Stall and Thayer, 1962; Marco and Stall, 1983; Cuppels and Elmhirst, 1999). Therefore, researchers looked for the alternatives that would resolve the resistance problems, be less harmful and would be effective to control the bacterial pathogens. Potentiality of many essential oils and their constituents have been tested against several plant pathogenic bacteria using different methods like agar dilution, disk diffusion, agar well and broth dilution assays (Perricone et al., 2015), however, regarding the *Mentha* essential oils there are few literature available from past to recent. Essential oils from *Mentha piperita* collected from different provinces like Turkey, Yakima, Mari-Linn Farms, and A Erdogmus Perfume Industry imported from India exhibited stronger antibacterial activity against *P. syringae* pv. *syringae, Pseudomonas syringae* pv. *tomato, P. syringae* pv. *phaseolicola, Xanthomonas campestris* pv. *campestris*, and *X. campestris* pv. *phaseoli* at 0.07–1.25 mg/ml range of MIC values (Iscan et al., 2002) by broth dilution bioassay. They also reported that some chemical constituents such as menthol and menthone derived from *M. piperita* essential oil showed potent MIC value against the plant pathogenic bacteria. Menthol (Polarome, Jersey City, NJ, United States) showed 0.625 mg/ml MIC value against *X. campestris* pv. *phaseoli*, 1.25 against *Ps. syringae* pv. *phaseolicola*, 0.07 against *Ps. syringae* pv. *tomato* and 0.156 against *Ps. syringae* pv. *syringae* and *X. campestris* pv. *campestris*. Similarly, (-)-menthone (Fluka, Germany) inhibited the growth of *X. campestris* pv. *phaseoli, P. syringae* pv. *phaseolicola, P. syringae* pv. *syringae* at 2.5 mg/ml MIC value, while of *X. campestris* pv. *campestris* and *P. syringae* pv. *tomato* at 1.25 mg/ml. Similar investigation was carried out by Soltani and Aliabadi (2012), they reported that essential oil from *M. piperita* and *M. spicata* showed same level of toxicity against *X. campestris* pv. *juglandis*. Vasinauskienë et al. (2006) evaluated seven essential oils including *M. piperita* and found that this oil is effective against phytopathogenic bacteria with zone of inhibition (ZOI) 2–6 cm against *P. syringae* pv. *syringae, P. syringae* pv. *tomato, Erwinia carotovora* subsp. *carotovora* and 6–12 cm against *X. vesicatoria* in disk diffusion bioassay. Minimum bactericidal concentration of *M. spicata* essential oils varied from 6 to 40 mg/ml in micro agar broth dilution method against *Acidovorax avenae* subsp. *citrulli* (Aac) causing seed borne bacterial disease in different commercially important crops. In another investigation, 1 μl dose of *M. arvensis* essential oil was more effective than streptomycin and showed a variable range of ZOI against *Pantoea agglomerans* (11.10 cm), *Erwinia amylovora* (10.9 cm), *Pantoea dispersa* (8.8 cm), *P. fluorescens* (5.2 cm), and *Ps. syringae* pv. *syringae* (5.0 cm) (Kokoskova et al., 2011). From the above investigations we can speculate that a variable range of efficacy was reported for different species of *Mentha* oil against the same bacterial species. This may be due to the use of different methodology during the screening, variability in bacterial strains and also variation in the chemical constituents of the essential oils of different *Mentha* species. The significant antibacterial activity observed for the different species of *Mentha* oil is due to the presence of phenolic compounds like menthol and carvacrol (Sivropoulou et al., 1995; Dorman and Deans, 2000). These phenolic compounds form complexes with bacterial enzymes and protein and inhibited the growth of bacterial pathogens (Rhouma et al., 2009). In the bacterial membrane phenolic compounds dissolved through penetration and where they interact with the cell metabolism cause the disruption of plasma membrane which increase the permeability and depolarize its potential finally led to death of bacteria (Oussalah et al., 2006; Xu et al., 2008).

### *Mentha* Essential Oil as Antifungal Agent

In the agriculture, fungi are the major plant pathogen in the field crops and storage food commodities (Sharifi-Rad et al., 2018), where by producing mycotoxins and several toxic metabolites they affect the nutritional value of food and make them unhealthy for human consumption (Paranagama et al., 2003; Sonker et al., 2015). Fungi reported to infect the plants in the field and storage include the genera of *Aspergillus, Cladosporium, Fusarium, Penicillium, Alternaria, Macrophomina, Rhizoctonia, Colletotrichum, and Botrytis* (Pandey et al., 2017), they are responsible for the 40–50% loss of the crop produce. For the management of these pathogens several synthetic fungicides like carbendazim, capton, and mancozeb are available in the market, however, these fungicides have their own limitations, use of such fungicides cause several side effect to the host as well as beneficial microorganisms. Also due to climate change some fungal pathogens has started to develop the resistance against the commercial fungicides (Chang et al., 2007; Price et al., 2015). Therefore, researchers were use the botanical fungicides for the management of these fungal pathogens which have broad range of toxicity, eco-friendly and renewable in nature. During recent years several essential oils have been proved as effective fungitoxicant against these pathogens including *Mentha* essential oils (Iacobellis et al., 2005; Teixeira et al., 2012; Goudjil et al., 2016). For the screening of essential oils against fungal pathogens basically few methods such as poison food method (Mishra et al., 2013), inverted Petri plate method (Pandey et al., 2013) and agar dilution methods (Vishvakarma et al., 2015) have been used. Poison food method was used to develop contact fungicides, while inverted Petri plate method was used to develop fumigant fungicides against the fungal pathogens. *Mentha longifolia* oil was fungistatic against *Aspergillus niger, A. versicolor, Cladosporium fulvum, Fusarium tricinctum, F. sporotrichioides, Penicillium funiculosum, P. ochrochloron* at 2.5 μl/ml and against *C. cladosporioides* at 12.5 μl/ml (Mimica-Dukic et al., 2003). The MIC of *M. piperita* varied from 1.13 to 2.25 mg/ml and 2.25 to 4.5 mg/ml for *P. digitatum, A. flavus, A. niger, Mucor* spp, and *F. oxysporum* (Tyagi and Malik, 2011). The essential oil and their chemical constituent’s like menthone and β-caryophyllene showed a different degree of inhibition against twenty-five microorganisms with more efficacy against *A. niger* (Marotti et al., 1994). Džamić et al. (2010) reported that 10 μl/ml of *M. longifolia* essential oil showed fungicidal activity against *Aspergillus* and *Fusarium* species, *P. funiculosum, Trichoderma viride* and 2.5 μl/ml to *C. fulvum, C*. *cladosporioides*, and *P. ochrochloron*. A variable range of inhibition zones (11–32, 19–30, and 16–29 mm) were reported for *M. longifolia, M. piperita*, and *M. spicata* essential oils, respectively, against *Rhizopus solani, A. niger*, and *Alternaria alternata* infecting stored food commodities. Correspondingly, these oils showed 44.1–157.8, 52.9–130.1, and 53.2–133.1 μg/mL respective range of MICs values against these fungal strains (Hussain et al., 2010a). Our laboratory bioassay revealed that essential oil of *M. arvensis* was found to be inhibited the growth of postharvest fungi of pigeon pea seeds and papaya fruits especially *Aspergillus* and *Fusarium* species (Singh, 2010; Pandey and Tripathi, 2011). However, on the contrary, results of Bouchra et al. (2003) showed that *M. pulegium* oil had moderate activity against *Botrytis cinerea* at 250 ppm where only 58.5% growth inhibition was reported. This differentiation in the toxicity may be due to the oils used from different plants and also it depends on the fungal strains of different hosts. *M. piperita* exhibited fungicidal nature against *Aspergillus* strains at 0.5–4 μl/ml (Saharkhiz et al., 2012). Fungal pathogens such as *Geotrichum citri-aurantii, P. digitatum*, and *P. italicum* causing fruit decay in citrus were controlled when treated with 750 μl/l dose of *M. spicata* oil (Regnier et al., 2014). Also formulation of essential oils has been tested for the control of fungal pathogens and they proved more effective than that of pure oil due to their formulation in adjuvant. The encapsulated oil of *M. piperita* was very effective at 800 ppm in controlling *A. flavus* causing postharvest rot in food commodities than that of pure oil which failed to caused complete mycelial inhibition at tested concentration range (upto 3000 ppm) (Beyki et al., 2014). *M. piperita* oil is also found to be effective against tomato pathogen *F. oxysporum* f.sp. *lycopersici* causing wilting with MIC of 0.3 μl/ml of air and at increased MFC, i.e., >0.6 μl/ml of air (Djordjevic et al., 2013). This oil has also been reported as effective antifungal agent against few soil borne pathogens *Drechslera spicifera, F. oxysporum* f.sp. *ciceris*, and *Macrophomina phaseolina* at effective concentration 1600 ppm by agar dilution method (Moghaddam et al., 2013). Some *Mentha* oils showed poor efficacy and were reported as phytotoxic in nature. In this regard, Lopez-Reyes et al. (2013) reported that *M. arvensis* oil (10% concentration) was poor antifungal agent in controlling *B. cinerea* and *M. laxa* growing on apricots and oil was also phytotoxic. Also, in their earlier research (Lopez-Reyes et al., 2010), 1 and 10% essential oil emulsion of *M. arvensis* was poor effective than that of others oils and fungicide tebuconazole in controlling fruit rot in apple due to *B. cinerea* and *P. expansum*. Similar results were reported for the fungitoxicity of *M. arvensis* oil against *F. oxysporum* causing wilt in crops (Gupta et al., 2011) at 10 and 20% oil concentration. In macro and microdilution methods, *M. spicata* essential oil exhibited 1.0–2.5 μl/ml range of MIC value in ethanol and 0.5–1.5 μl/ml in Tween against plant pathogens namely *A. niger, A. ochraceus, A. versicolor, A. flavus, A. terreus, A. alternata, P. ochrochloron, P. funiculosum, C. cladosporioides, T. viride, F. tricinctum*, and *Phomopsis helianthi*. However, MICs of *M. piperita* essential oil were higher, 1.5–3.0 μl/ml in ethanol and 1.0–2.5 μl/ml in Tween against the same pathogens (Soković et al., 2009). These both oils, i.e., *M. spicata* and *M. piperita* also performed significant antifungal activity against major pathogens of button mushroom, i.e., *Verticillium fungicola* and *T. harzianum* (Sokovic and van Griensven, 2006) by micro and macro-dilution methods. *M. spicata* oil was fungistatic at 0.5–2.5 μl/ml, while fungicidal at 1.5–2.5 μl/ml. While MIC (2.5–3.5 μl/ml) and MFC (3.0–4.0 μl/ml) values of *M. piperita* oil (Sokovic and van Griensven, 2006) increased by micro and macro-dilution methods.

Chemical constituents from essential oils from *Mentha* species has also been reported as potential antifungal agent against plant pathogenic fungi. Monoterpenoid, i.e., spearmint gave 100% mycelial inhibition of postharvest fungi *A. terreus* and *F. oxysporum*, and 91% of *P. expansum* and *V. dahliae* at 1 μl/l dose and also at the same dose this terpenoid inhibited 100% conidial production of all these tested fungi (Kadoglidou et al., 2011). Menthol extracted from *M. spicata* showed MICs of 0.25–1.5 μl/ml in ethanol and 0.05–1.0 μl/ml in Tween by microdilution method (Soković et al., 2009), while carvone of *M. piperita* possessed higher antifungal activity with MICs value 0.25–1.0 μl/ml in ethanol and 0.05–0.5 μl/ml in Tween by the same method. Limonene showed moderate fungistatic activity against aforesaid pathogens with MICs by microdilution method were 6.0–11.0 μl/ml in ethanol and 5.0–9.0 μl/ml in Tween (Soković et al., 2009). This variation in MICs of *M. spicata* and *M. piperita* may be due to the variation in chemical ingredients among the plant species, methodology used and also different fungal strains used during the bioassays. The greater efficacy of *M. piperita* and *M. spicata* reported were due to the presence of menthol and carvone. This antifungal activity of essential oil may be due to presence of oxygenated terpenes or phenolic structure. This is speculated that the hydroxyl group of phenol and alcohol might be an important factor of their antifungal activity (Griffin et al., 2000).

Researchers reported that several monoterpenes acts on cell membrane by affecting lipid fraction of plasma membrane, causing leakage of intracellular membrane (Trombetta et al., 2005). Monoterpenoids are also found to affect respiratory enzymes of fungi (Cox et al., 2000), inhibited the uptake of microbial oxygen and oxidative phosphorylation. This could be a reason why some essential oils rich in monoterpenic components were found to be effective against the plant pathogenic fungi. These all results on the use of essential oils and their chemical constituents of *Mentha* species revealed that these oils could find practical application in the prevention and protection of fungal infections of plants in the field as well as in the storage conditions.

### *Mentha* Essential Oils as Repellent, Insecticidal, Antifeedant Agents Against Stored Insect Pests and Its Mechanism of Action

In the tropical countries insects are the major destroyers of stored food commodities especially of cereals and pulses. The important storage insect pests such as pulse beetles (*Callosobruchus* species), maize weevil (*Sitophilus zeamais*), rice weevil (*S. oryzae*), and red flour beetle (*Tribolium* species) are reported to cause about 60% loss in cereals and pulses (Singh et al., 2012). These problems arise due to agroclimatic conditions as well as improper storage facilities. In recent years, there are several chemical and non-chemical methods were used for the control of these insect pests. Use of commercial fumigants against these stored insect pests is either effective, but due to climate change insects have started develop resistant against these fumigants (Chaudhry, 1997) or also these insecticides have several side effect on human beings. Use of essential oils is one of the non-chemical options for the management of these insect pests which have been proved as effective method for the management of stored product insects and also have fewer chances of resistance problems against these insect pests. Earlier, Kumar et al. (2010) reviewed the insecticidal properties of extract and essential oils from different *Mentha* species against storage as well as field insects’ pests. In continuation of these reports, here we are updating the report on 2000 onwards, dealing only on the efficacy of essential oils against stored insect pests. Attempts have been made to determine the insecticidal efficacy of essential oils and their constituents from different *Mentha* species against stored product insects. *Mentha haplocalyx* (31.5 μg/cm^2^) essential oil at 72 h of exposure showed 83% repellent activity against *T. castaneum* when assessed by area preference method (Wagan et al., 2016). *M. haplocalyx* essential oil and its main constituent’s menthol, menthyl acetate, limonene, and menthone had LD_50_ values of 16.5, 7.91, 5.96, and 13.7 g/adult, respectively, against *Lasioderma serricorne* adults in contact toxicity bioassay and among all menthol showed higher repellent activity (Zhang et al., 2015). In fumigant toxicity test, *M. spicata* showed 27.52 μl/l of air LC_50_ values against *Rhyzopertha dominica* adults affecting stored maize (Nubia et al., 2016). However, in their study this oil was reported as poor fumigant than that of *Ocimum basilicum* oil. However, *M. spicata* essential oil exhibited 100% mortality to *C. chinensis* during a fumigation test with an LC_50_ value of 0.003 μl/ml air after 24 h of treatment and 100% repellency at 0.025 μl/ml air concentration. Oil showed 98.46% oviposition deterrence, 100% ovicidal activity, 88.84% larvicidal activity, 72.91% pupaecidal activity, and 100% antifeedant activity against *C. chinensis* (Kedia et al., 2014). *M. spicata* oil showed a more than 80% mortality of *Ephestia kuehniella* (Zeller) and *Plodia interpunctella* (Hubner) at 2.5 ml/l dose and 2 h of exposure times (Eliopoulos et al., 2015). They found that oil caused 50–60% of egg mortality, and 18 and 28% of larval and pupal mortality, respectively, at same dose and 24 h of exposure. In *T. castaneum, M. arvensis* oil was found to reduce 67.50 and 61.25% acetylcholinesterase activity over control (Mishra et al., 2014) after 24 h of fumigation. Saroukolai et al. (2014) reported that *M. spicata* essential oil showed 259.73 and 75.31 ppm LC_50_ value against fourth star larvae of potato beetle *Leptinotarsa decemlineata* (Say) by fumigant bioassay and oil also exhibited 39.26% feeding deterrent index against the adults at 16 ppm. *M. pulegium* essential oil and its major component, pulegone, showed potent insecticidal activity against mushroom scatopsid flies, *Scatopse* spp. (after 0.5 h, LC_50_ = 0.17 and 0.13 μl/L air, respectively) in fumigant bioassay and 100% mortality of adults were observed at 4 h of exposure period (Gurkan and Fedai, 2013). In another study, adults of *T. castaneum* and *C. maculatus* were killed by *M. longifolia* essential oils at 13.05 μl/l air LC_50_ value by fumigant bioassay (Abbas and Javad, 2012). Halit et al. (2012) studied the fumigant effect of three essential oils of the genus *Mentha* such as *M. spicata, M. villoso-nervata*, and *M. piperita* against *S. granarius*. Among these *M. villoso-nervata* exhibited 90% mortality of adults by fumigant bioassay, while its main constituent carvone exhibited 100% mortality at 24 h of exposure with 0.024 μl/ml LC_50_ value. Thus, *M. villoso-nervata* and carvone can be commercialized as potent insecticidal agent against *S. granarius*. Leaf essential oil of *Mentha* species possessed 55% mortality after 48 h of exposure with LD_50_ values 0.044 μl/ml by topical application and 3.51 μl/cm^2^ by fumigant application against the *T. castaneum* adults (Franz et al., 2011). Author reported that this oil is poor insecticidal agent than that of *Cymbopogon citratus*. It was found that 1.75 μl of essential oil per 0.5 ml acetone dose of *M. viridis* strongly repelled (63 81%) *S. granarius* adults, thus this oil can be used in organic food production as a repellent and insecticidal agent (Somaye, 2010). Also 100% mortality of *S. granarius* was achieved by *M. spicata* subsp. *tomentosa* and *M. spicata* var. *formasa* essential oils at 1 μl/l air and the exposure periods of 36 and 48 h and potent mortality of adults at 0.5 μl/l air and an exposure period of 48 h (Irfan et al., 2009). In the study of Mohamed and Abdelgaleil (2008) *M. microphylla* exhibited potent fumigant activity against *T. castaneum* (LC_50_ = 4.51 μl/l) and *S. oryzae* (LC_50_ = 0.21 μl/l). Thus, this oil can be used as protectant against this insect. *M. longifolia* essential oil exhibited 100% mortality of *S. zeamais* deteriorating maize at 0.50 μl/g dose in contact toxicity bioassay with 100% repellency at the same dose (Odeyemi et al., 2008). *M. viridis* essential oil showed more that 50% mortality of *C. maculatus* at 235 ppm dose of essential oil and also reduced 67.4% egg hatching and 72% of progeny emergence at 300 ppm dose (Derbalah and Ahmed, 2011). Inhibition of oviposition in insects by essential oils is also an important criterion to manage the stored product insects. There are several essential oil including *Mentha* are reported as effective ovipositional inhibitor against eggs of stored product insects. Kumar et al. (2009) reported that essential oil from *Mentha* species is very effective and completely inhibited oviposition of *C. chinensis* at 200 μl/l and protected seed (94.05%) from biodeterioration of *C. chinensis*. Against adults of *C. maculatus, Mentha* oil showed 4.43 μl/l air LC_50_ values and at 1.01 μl/l air dose possessed anti acetylcholinesterase activity (Al-Sarar et al., 2014).

**FIGURE 2:**
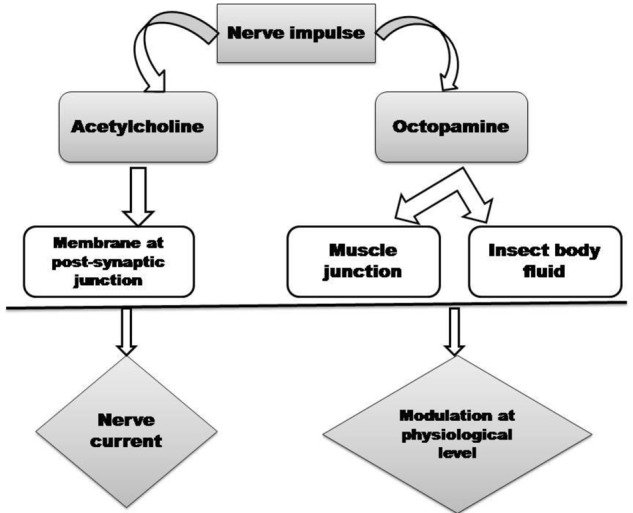
Target sites as promising neurotransmitter mediated toxic action of essential oils in insects.

We have described here that insecticides based on essential oil are the good options for the management of insect pests hazardous and resistance problems of the commercial insecticides. To combat with increasing resistance rate in commercial insecticides, identification of novel effective insecticidal compounds is essential. Researchers reported that essential oil based botanical insecticides have wide range of target action on insect pests. When insects are exposed to the essential oils, breakdown of the nervous system of insects occurs (Kostyukovsky et al., 2002). The main target sites of essential is octopaminergic system (**Figure 2**) which plays a key role as a neurotransmitter, neurohormone, and neuromodulator in invertebrate systems, with a physiological role analogous to norepinephrine in vertebrates (Shaaya and Kostyukovsky, 2006). During the insecticidal activity of the essential oils, the mechanism behind the insect mortality is that the volatiles penetrates in the insect body via respiratory system and results in abnormal breathing which leads to asphyxiation and final death of insects (Pare and Tumlinson, 1999). Some investigators also reported that acetylcholinesterase enzyme activity in insect is also inhibited by the essential oil and constituents (Picollo et al., 2008) which lead to the blockage of nerve impulse, later paralysis and then death of the insects occurs. Miyazawa et al. (1998) reported that three *Mentha* oils viz., *M. aquatica, M. gentilis*, and *M. arvensis* essential oils significantly inhibited acetylcholinesterase (AChE) activity and their IC_50_ values were in the range of 28-32 μg/ml. *Mentha* essential oils also showed oviposional activity against the stored insects. This may be due to either death of the insects before their egg laying, failure of live females to lay eggs when they come in contact with essential oils (Pandey et al., 2011). Since *Mentha* essential oils are made up of terpenic and phenolic chemical constituents, which affect the octopamine receptor and inhibit the acetyl cholinesterase of the larvae and pupae, thereby killing them and protect the food commodities (Khanikor et al., 2013). Additionally, multiplication of larvae and pupae do not occur inside the grains due to low penetration of essential oil vapors inside the grains, which further protect the grains from infestation of larvae and pupae (Rahman and Schmidt, 1999). Changes in physiology and behavior of insects are also affected by the essential oils which further affect the nervous system. These essential oils also cause reduction in egg laying capacity when it comes in contact with adults as described in the above section. Due to these properties essential oils are also addressed as semiochemicals and have been implemented in IPM (integrated pest management) program in place of those which cause lethality to insects (Rajendran and Sriranjini, 2008). Investigators also reported that in few studies eggs were laid by insects but progeny emergence failed due to the potent components of the oils which penetrate into the eggs via chorion and inhibit the embryonic development (Abdullahi et al., 2011). Failure of egg hatching is also occur when essential oils interferes the physical process of eggs, causing alteration in oxygen and surface tension within the eggs (Abdullahi et al., 2011). Therefore, at beginning of the lifecycle, the population of insects can be reduced by inhibiting their ovipositional behavior. In this paper we reviewed that *Mentha* oil from different species also caused inhibition of egg laying and progeny emergence of several stored product insects. Therefore, these *Mentha* oil based ovipositional inhibitors would be useful against insects developing resistance treated with those responsible for lethal toxicity. Such properties of *Mentha* oil strengthen their recommendation in storage system due to infestation caused by insects. *Mentha* oils also have ovicidal activity. The mechanism behind ovicidal property is that, through the posterior pole the vapors of essential oil circulate into the eggs and disrupt embryonic development of eggs causing death of the embryo (Credland, 1992). Additionally, essential oils exhibited ovicidal action, may act as neurotoxins when development of nervous system starts (Papachristos and Stamopolos, 2002). It is also reported in this paper that researchers were screened essential oils of *Mentha* species at different life stage of the insects. Hence, during the insecticide formulation the *Mentha* oil exhibited potent toxicity at all stages should be considered.

## Conclusion and Constraints

The compiled review evidenced that different species of the genus *Mentha* possessed essential oils which have a wide range of differences in their chemical constituents in samples collected from the different countries. The major components reported in the essential oils of the genus *Mentha* were pulegone, menthon, menthol, carvone, 1, 8-cineole, limonene, β-caryophyllene. Menthol is the major derivative product of *Mentha* species and is widely used in pharmaceuticals, tobacco products, perfumery, aromatherapy, toothpastes, soaps, cosmetics, oral preparations, confectionaries and even in cigarettes. *Mentha* oil and its derivatives is currently used industrially for various purposes in form of menthol crystals, dementholized oil (DMO), L-menthol, menthone, natural crude *Mentha* oil, *cis* 3 hexenol, menthyl acetate, piperitone, limonene, menthofuran, and spearmint terpene. Most commercialized species of *Mentha* are *M. arvensis, M. piperita*, and *M. spicata.* Review also showed that essential oils and chemical constituents from the different species of the genus *Mentha* are very effective in controlling the fungal and bacterial plant pathogens as well as stored product insects like *Callosobruchus* and *Tribolium* species.

Many essential oils have proven their effectiveness as a repellent agent against many storage pests as well as other arthropods including mosquitoes. But directly using essential oils for pest control has some shortcomings viz., volatility, short shelf life and regulatory issues for disbursing it freely in environment. Thus, more insight is needed to overcome the barriers of oil use as a pest control agent like, exploring fixative materials for sustained release, application methods and protocols, managing environmental issues, residual phytoxicity, overcoming toxicological and regulatory barriers. One of the promising aspects of use of *Mentha* oil as grain/food crop protectant is that its favorable mammalian toxicity because its constituents are already used in several products for human consumption. Thus, ecofriendly, biodegradable plant based pesticides which are already in conventional use need less time for their commercial launch in market.

*Mentha* oil is already used commercially, international products like EcoSMART^®^-ant and roach killer and mosquito repellent uses peppermint oil (1.5%). It is also one of the constituents in flea shampoos, mosquito repellents topical preparations. Thus when the current human population is now sensitized against use of several harmful xenobiotics as pesticides causing deep environmental and health hazards, switching to plant based alternatives is already on the way and these bio product based industry will strengthen in recent future.

Still there are constraints for *Mentha* oil and its derivatives as faced by other popular essential oils. High volatility decreases time of protection, thus it needs to be used as impregnated material with some other products. Effects on non-target microorganisms including pollinators, bees, and natural predators needs to be evaluated. There are bottle necks for its wide use, as not all natural products are always safer and learning from our past, rampant use of chemical without assessing its long term effects would be a mistake. Thus, natural products must be scientifically validated for their long term use and release. Issues regarding its formulation like proper identification, pressure on natural resources, phytotoxicity, mammalian toxicity, standardization of product, registration and regulation have to be taken care of for its overall commercialization.

Further most promising natural products can pave a way for their synthetic manufacture which will pose less pressure on natural resources and will be economically feasible for their wide spread use as a pesticide. Thus, antimicrobial and insecticidal properties of essential oils of *Mentha* species offer the prospect of using them as natural pesticides and they can have market niches with a commercial value. Finally, a systematic and gradual approach for embracing natural products against several pests of agriculture without jeopardizing commercial, social interest is need of the day for a healthy sustainable environment in future.

## Author Contributions

AP contributed as co-author in reviewing the literature, compiling the information, preparing the review draft, and revising the manuscript. PS guided co-author to outline the sections, compile the manuscript, critically reviewed and revised the manuscript, and restructured the entire manuscript with significant contribution to shape the manuscript for the final version.

## Conflict of Interest Statement

The authors declare that the research was conducted in the absence of any commercial or financial relationships that could be construed as a potential conflict of interest.
